# Prevalence, Variability and Bioconcentration of Saxitoxin-Group in Different Marine Species Present in the Food Chain

**DOI:** 10.3390/toxins9060190

**Published:** 2017-06-12

**Authors:** Javiera Oyaneder Terrazas, Héctor R. Contreras, Carlos García

**Affiliations:** 1 Physiology and Biophysics Program, Faculty of Medicine, University of Chile, Santiago 8380000, Chile; joyaneder@live.com; 2 Department of Basic and Clinical Oncology, Faculty of Medicine, University of Chile, Santiago 8380000, Chile; hcontrer@med.uchile.cl

**Keywords:** saxitoxin group, risk assessment, shellfish, fish, LC-PCOX, Chile

## Abstract

The saxitoxin-group (STX-group) corresponds to toxic metabolites produced by cyanobacteria and dinoflagellates of the genera *Alexandrium, Gymnodinium*, and *Pyrodinium*. Over the last decade, it has been possible to extrapolate the areas contaminated with the STX-group worldwide, including Chile, a phenomenon that has affected ≈35% of the Southern Pacific coast territory, generating a high economic impact. The objective of this research was to study the toxicity of the STX-group in all aquatic organisms (bivalves, algae, echinoderms, crustaceans, tunicates, cephalopods, gastropods, and fish) present in areas with a variable presence of harmful algal blooms (HABs). Then, the toxic profiles of each species and dose of STX equivalents ingested by a 60 kg person from 400 g of shellfish were determined to establish the health risk assessment. The toxins with the highest prevalence detected were gonyautoxin-4/1 (GTX4/GTX1), gonyautoxin-3/2 (GTX3/GTX2), neosaxitoxin (neoSTX), decarbamoylsaxitoxin (dcSTX), and saxitoxin (STX), with average concentrations of 400, 2800, 280, 200, and 2000 µg kg^−1^ respectively, a species-specific variability, dependent on the evaluated tissue, which demonstrates the biotransformation of the analogues in the trophic transfer with a predominance of α-epimers in all toxic profiles. The identification in multiple vectors, as well as in unregulated species, suggests that a risk assessment and risk management update are required; also, chemical and specific analyses for the detection of all analogues associated with the STX-group need to be established.

## 1. Introduction

The saxitoxin-group (STX-group) corresponds to polar chemical compounds produced by cyanobacteria and dinoflagellates of the genera *Alexandrium*, *Gymnodinium*, and *Pyrodinium*, which under natural conditions produce harmful algal blooms (HABs). The STX-group is made up of neurotoxins with high affinity and voltage-dependent sodium channels that cause muscle paralysis by blocking the nervous impulse [1,2]. Up to now, over 57 isomers related to this group have been identified, each of them possessing different toxic capabilities. These toxins are constituted by a unit called imidazoline that, according to the modification of some of its functional groups, can be divided into three groups: non-sulfated-carbamate toxins, 11-hydroxysulfated-carbamate, and 21-N-sulfocarbamoyltoxin [3]. Their toxic effects are related to the type of analogue involved in HABs, each characterized by having a different toxicity (toxic equivalent factor, TEF). Thus, toxicity is related according to the groups characterized in a descending way i.e. non-sulfated-carbamate toxins > 11-hydroxysulfated-carbamate > 21-N-sulfocarbamoyltoxin [4,5].

The most important toxic analogues detected in marine products are gonyautoxins (GTX3/GTX2, dcGTX3/dcGTX2, GTX5, and GTX4/GTX1), neosaxitoxin (neoSTX), decarbomoylsaxitoxin (dcSTX), and saxitoxin (STX) [6]. Aquatic organisms (bivalves and tunicates) are characterized by a high clearance rate; therefore, they accumulate high concentrations of toxins in their tissues, which turn some species into excellent HABs bioindicators [7]. In addition, all these organisms are highly valuable for their texture, flavor, and chemical and nutritional properties, making them products with a high commercial value [8]. In recent times, geographical expansion of HABs worldwide has been recurrent, which has been associated with climate change, since their impact on the ocean causes a change in the marine phytoplankton community, resulting in spatial and temporal expansion or contraction of HABs. However, it all depends on the interaction of toxic cell communities within the new environment which they will inhabit [9,10,11].

The export of mussels represents an important economic contribution to Chile with annual figures of approximately US$1424 million, among frozen (91.6%) and canned products (8.4%); the main countries that import these resources are Spain (25.4%), France (15.1%), and the USA (12.2%). In addition, other export products in recent years have acquired great commercial relevance, such as crabs, red sea urchins, clams, ribbed mussels, top shells, and loco [12].

The current international regulatory standard states that shellfish must have levels ≤80 µg STX equivalent 100 g^−1^ in which the mouse bioassay (MBA) is the official method for their detection [6,13]. Notwithstanding, a number of countries have begun to use alternative analytical methods, such as Pre-COX-LC-FLD (Lawrence method) [14], LC-PCOX [15] or LC-MS/MS, to determine and quantify each of the toxic analogues associated with the STX-group [16,17].

HABs have been constant in the last 40 years in the Southern Pacific (Chile), causing great economic damage; in the year 2016 alone, losses were estimated to be ≈US$200 million, with a period length of approximately 10 months. For this reason, constant monitoring programs have been developed to identify STX-producing microalgae at a national level, in order to establish, in a timely manner, the total toxicities in contaminated seafood, such as in mussels (*Mytilus chilensis*), clams (*Venus antiqua*), and gastropods (*Concholepas concholepas*) [18,19].

The risk of exposure of people to STX-group toxins is directly related to the consumption of marine foods (shellfish and sea products) contaminated with STX-group toxins, which may lead to signs of severe poisoning with symptoms such as tingling sensations in the lips, mouth, and tongue, numbness of extremities, parestesias, weakness, ataxia, floating/dissociated feeling, nausea, shortness of breath, dizziness, vomiting, headache, dysphagia, and dysartheria [20,21]. Symptoms begin between ≈15–60 min after contaminated shellfish are ingested; the time depends on the toxicity of the shellfish (≈120 µg STX equivalent per person), which can result in the death of people between 1–4 h post-intoxication (400–10,000 µg STX equiv per person), which is caused by asphyxiation [22,23].

Fatality rates from the STX-group range between 1–12% in an isolated outbreak. The high mortality rates in some areas are almost certainly caused by poor access to advanced life support [24,25]. However, certain age groups (<14 years and >65 years) may be at high risk when overexposed to the consumption of seafood with a toxicity of ≈37 µg STX equiv 100 g^−1^, a value considered as the limit in the detection of STX-group toxins by the MBA [6]. 

The information regarding the determination of toxic profiles and the distribution dynamics in different marine species from the Southern Pacific coast is scarce, as is the chemical variability of the different analogues related to the STX-group, which when assimilated are biotransformed by diverse marine organisms. Therefore, in the present paper, we have investigated: (a) the interspecific accumulation (concentration) and distribution (seafood tissue) of STX-group toxins in marine species (echinoderms, bivalves, algae, gastropods, crustaceans, tunicates, cephalopods, and fish) exposed to an associated bloom of *Alexandrium catenella* in the austral fjords of Chile; (b) the prevalence of STX-group toxins in species from different (rocky and sandy) substrates, and (c) we have assessed how much our daily intake of STX-group toxins is from the consumption of these different sea products.

## 2. Results and Discussion

The levels of toxins detected in the different assessed species were dependent on the type of sample, the collection sites, and the relation to the habitat where the species were collected. At least four factors can affect the concentration of toxins in seafood tissues: species, ecology (rocky strata, sandy and benthic bottom habitats), morphological parts (digestive glands, adductor muscle, mantle and foot) and physical properties of the water (nutrient level, pH, salinity, and temperature).

### 2.1. Prevalence of Alexandrium Catenella in the Study Area

**Figure 1:**
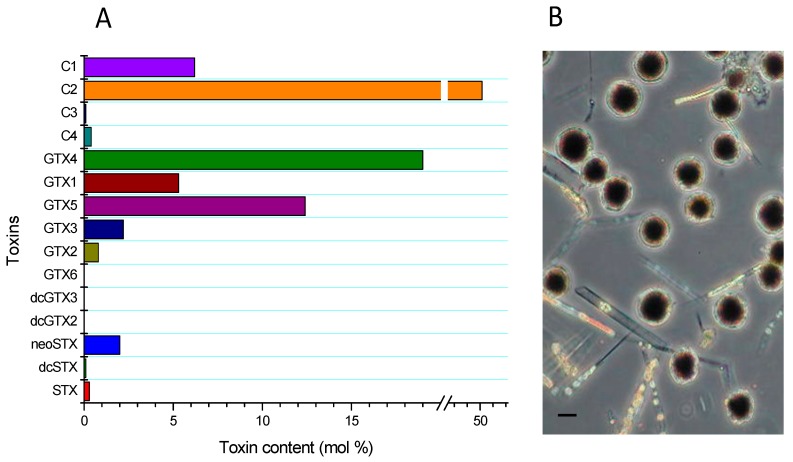
Toxin profile (**A**) of *Alexandrium catenella* and (**B**) phytoplankton sample collected at the Huichas Island in the Aysén Región. Scale bars = 10 μm.

The cellular levels detected in the area were scarce; notwithstanding, *Alexandrium catenella* blooms have been permanently described, and which tend to occur usually between January and March. It is worth noting that the influence of oscillation in the ocean affects both the general circulation characteristics of the interior waters and the characteristic of the water column (temperature), favoring or inhibiting the *Alexandrium catenella* blooms [26,27,28]. The prevalence of the detected *A. catenella* blooms is associated with average temperatures of 15 °C and a salinity of 35 psu, with a profile composed by 70% N-sulfocarbamoyl derivatives (C1/C2, GTX5), 24% carbamoyltoxins (GTX4/GTX1), and in smaller proportion (≈3%) GTX3/GTX2, neoSTX and STX; in trace levels dcGTX3/dcGTX2 and dcSTX may be present and a toxicity of ≈15 pg STX equiv cel^−1^ was detected with a profile characteristic of the area (Figure 1) [29,30]. Regarding the composition of the toxic profile in the different species of *Alexandrium* sp., variability is directly related to the season in which they are assessed. Thus, toxic profiles obtained in spring had predominantly (%mol) β-epimers (C2, GTX4, GTX3) and in autumn α-epimers (C1, GTX1, GTX2), this is, because, during periods unfavorable to blooms, toxins that form part of the profile tend to transform (epimerization) into more stable forms. The above mentioned is directly related to the data obtained from cysts, in which the profiles are predominated by forms of 11-α-hydroxysulfate epimer, clearly indicating that the formation of these epimers in cysts are correlated with nitrogen-limited states [31]. 

Variability in relative abundance of species in the different zones is limited to local environmental and hydrogeographic factors that tend to affect the strength and timing of sexual induction and, therefore, the process of germination or encystment of *A. catenella* [32]. Nevertheless, precautionary closures of areas for the identification of HAB and toxins associated with the STX-group do not involve prohibition of extraction of all species [18,19]. In addition, these areas have been characterized by the presence of cysts, which has been historically correlated with the abundance of cells [33,34,35]. The sexual cycle of *A. catenella* has been characterized by a dormant benthic stage, which is correlated with the dispersion of the species in the region and latitudes further north of the country (Chiloé island), which allows it to obtain a resistance to the unfavorable abiotic conditions of the region. In addition, its pelagic-benthic process in the zone allows it to maintain a prolonged period of low vegetative cell concentrations in the column water, favoring the accumulation (toxic/non-toxic cells) by the marine organisms present in the area [7,36,37]. 

### 2.2. Prevalence of STX-Group in Fresh Shellfish Samples

**Figure 2:**
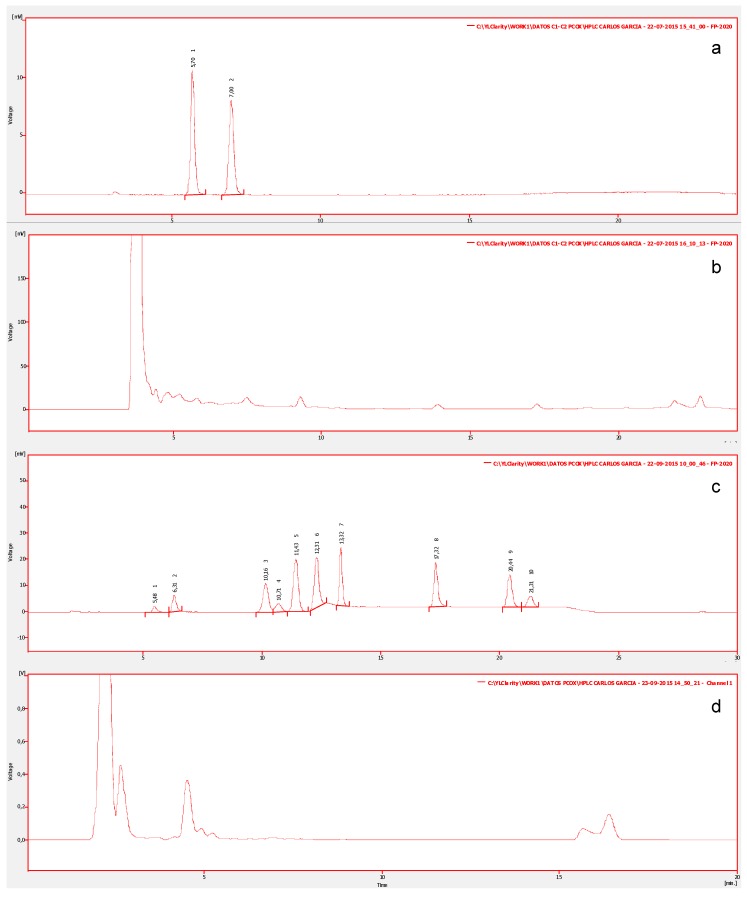
High Resolution Liquid Chromatography with Fluorescent Detection (LC-PCOX) chromatograms from shellfish extracts. Chromatogram (**a**) shows the certified reference material of the standards mix of group C1/C2. Chromatogram (**b**) shows non-visceral tissues of *Mytilus chilensis* to group C1/C2. Chromatogram (**c**) shows the standards mix of the carbamate group (GTXs/STX) corresponding to: GTX4/GTX1, dcGTX3/dcGTX2, GTX-5, GTX3/GTX2, neoSTX, dcneoSTX, and STX. Chromatogram (**d**) shows tissues of *Macrocystis pyrifera* with the carbamate group.

For this study, 150 samples of ten types of edible shellfish, collected from natural banks, were analyzed. In order to determine the profile of toxins, all species were analyzed to detect and identify STX-group toxins. Figure 2a shows the chromatograms of the certified reference material of the standards mix of group C1/C2 (N-sulfocarbamoylgonyautoxin-2/3) which shows two chromatographic peaks corresponding to: N-sulfocarbamoylgonyautoxin-2 (C1) and N-sulfocarbamoylgonyautoxin-3 (C2), with retention times (R_t_) of 05:70 and 07:00 min respectively. Additionally, the standards mix of the carbamate group (GTXs/STX) (Figure 2c), shows 10 chromatographic peaks corresponding to: gonyautoxin-4/1 (GTX4/GTX1), decarbamoylgonyautoxin-3/2 (dcGTX3/dcGTX2), gonyautoxin-5 (GTX-5), gonyautoxin-3/2 (GTX3/GTX2), neosaxitoxin (neoSTX), decarbamoylneosaxiton (dcneoSTX), and saxitoxin (STX), with R_t_ of: 05:48, 06:31, 10:16, 10:71, 11:43, 12:31, 13:32, 17:32, 20:44, and 21:32 min respectively. In all evaluated marine species, toxins with identical R_t_ as STX-group in their visceral and non-visceral tissues were detected. Some chromatographic peaks eluting at the beginning of the run, with R_t_ different from standard toxins, are associated with pigments within the matrix of invertebrates (Figure 2b,d). 

**Figure 3:**
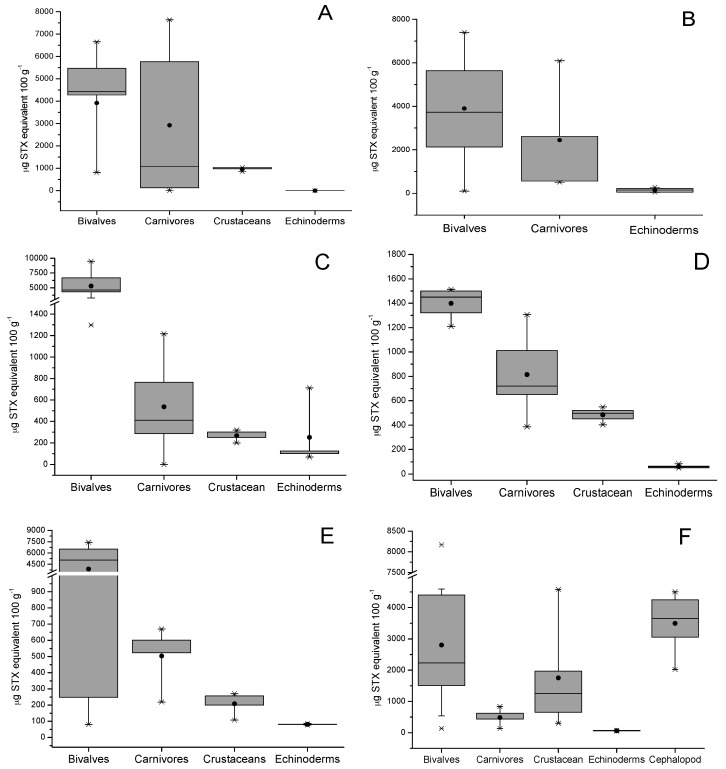
The saxitoxin-group (STX-group) toxin contamination in different shellfish species. (**A**–**F**) represent the different extraction areas of marine vertebrates (see Section 4). The bottom and top of the box are the first and third quartiles; the band inside the box is the second quartile (the median); the ends of the whiskers represent one standard deviation above and below the mean of the data, and the dots are mild outliers.

Additionally, the total toxicity of all toxins related to the STX-group in different species of shellfish are shown in Figure 3; the presence of this group of toxins in different species of shellfish (bivalves, gastropods, crustaceans, echinoderms, and fish) collected from six zones in the southern austral of Chile was detected (see Section 4). The average concentration of STX-group toxins in tested shellfish samples decreased in the following order: bivalves from rocky strata > bivalves from sandy bottom > gastropods > tunicates > echinoderms > cephalopods > fish. The data show that each zone showed maximum values for bivalves with a toxicity between 1500 to 10,000 µg STX-equiv 100 g^−1^, levels directly related to the high filtration capacity of this type of species (*Mytilus chilensis* clearance rate 2.39 ± 0.4 L h^−1^; *Tagelus dombeii*, clearance rate 0.79 ± 0.3 L h^−1^) [38] (Figure 3A–C). In relation to bivalves from soft bottoms, the abundance of resources was directly related to the areas with the highest salinity (zones 1 and 6), a factor that is directly associated with the increase of their metabolic rates to support greater osmoregulation and compensate by increasing their filter-feeding rate [39]. In gastropods, the detected toxicity ranged between 129.8 and 7644 µg STX-equiv 100 g^−1^; this toxicity is completely dependent on the toxic diet obtained in the sampling area (mussels consumed per day by *Concholepas concholepas* ≈0.18 to 0.69) [40] (Figure 3B). In relation to crustaceans, the toxicity detected ranged between 106.6 and 4583 µg STX-equiv 100 g^−1^, this group includes crabs (*Homalaspis plana*) whose toxicity is mainly related to the opportunistic diet (food intake of ≈3.19% body weight) and limited to the availability of food (small crustaceans, molluscs, echinoderms, polychaetes, fish, and algae) [41] (Figure 3E). In this group, the omnivorous filter feeder, giant barnacle (*Austromegabalanus psittacus*, clearance rate 1.26 L h^−1^) is included; in this species, an average toxicity of 301.1 µg STX-equiv 100 g^−1^ was detected and its diet is exclusively related to planktonic microalgae (Figure 3C) [42,43]. 

The toxicity dynamics also included echinoderms such as red sea-urchin (*Lexochinus Albus*), in which a toxicity between 0–186.3 µg STX-equiv 100 g^−1^ was detected; this species stands out for being of great economical importance in Chile (Figure 3C) [44] and its diet is mainly brown algae (*Ulva* sp. and *Macrocystis* sp. ≈45 g d^−1^); therefore, its toxicity would be related to grazing, a process by which it captures toxins from the sediment and from pseudofeces coming from other species (bivalves). This group included the assessment of sea stars (*Stichaster striatus*), and *Fissurella nigra*. In sea stars, a toxicity between 39.7 and 135.3 µg STX-equiv 100 g^−1^ was detected, assimilating the said toxicity through its diet related to the consumption of clams (0.50 ind d^−1^) and blue mussel (0.27 ind d^−1^) [45]. Additionally, in *Fissurella nigra*, a toxicity with a value close to 15 µg STX-equiv 100 g^−1^ was detected; it is a herbivorous species whose main diet is *Ulva* sp. characterized by its role of grazers in controlling primary production [46]. 

Tunicates (sea squirts, zone 4, Figure 3B), which are characterized by a diet related to benthic suspended foods and pseudofeces from other species, a shown toxicity of ≈420 µg STX-equiv 100 g^−1^ (clearance rate 1.29 L h^−1^). In echiurans (*Urechis chilensis*, spoon worms), sediment feeders, and detritus feeders, levels of ≈714.2 µg STX-equiv 100 g^−1^ were detected (clearance rate 6–13 kg m^−2^ year^−1^) [47]. 

In the study area, the evaluation considered two final vectors of the food chain, cephalopods and fish. In cephalopods (*Enteroctopus megalocyathus*) captured in zone 6, a toxicity of ≈3100 µg STX-equiv 100 g^−1^ was detected (Figure 3F), which results from the fact that it is a carnivorous species whose diet mainly consists of small fish, crustaceans, and molluscs, from which it assimilates toxins [48]. Finally, the evaluation of fish in culture (Atlantic salmon, *Salmo salar*) detected STX-group toxins only in the viscera at levels <LOD, where toxins in other tissues (muscle) were not detected. 

Considering the high biotransformation rate of aquatic species and the interconversion of analogues into thermodynamically more stable molecules, it is possible to detect and identify at least seven toxic analogues in each species: GTX4/GTX1, GTX3/GTX2, neoSTX, dcSTX, and STX, in concentrations ranging from 2.5 to 5357 µg 100 g^−1^. The STX-group has been consistently identified in the southern coast of Chile, but its identification has been limited to the determination of toxicity in bivalves through MBA, with few data related to the profile of the identified toxins and to their relationship among the toxic analogues detected in different aquatic species [7]. It should be noted that it has even been possible to detect simultaneous occurrence with other toxic groups, such as the YTX-group and the OA-group [7,49]. 

**Figure 4:**
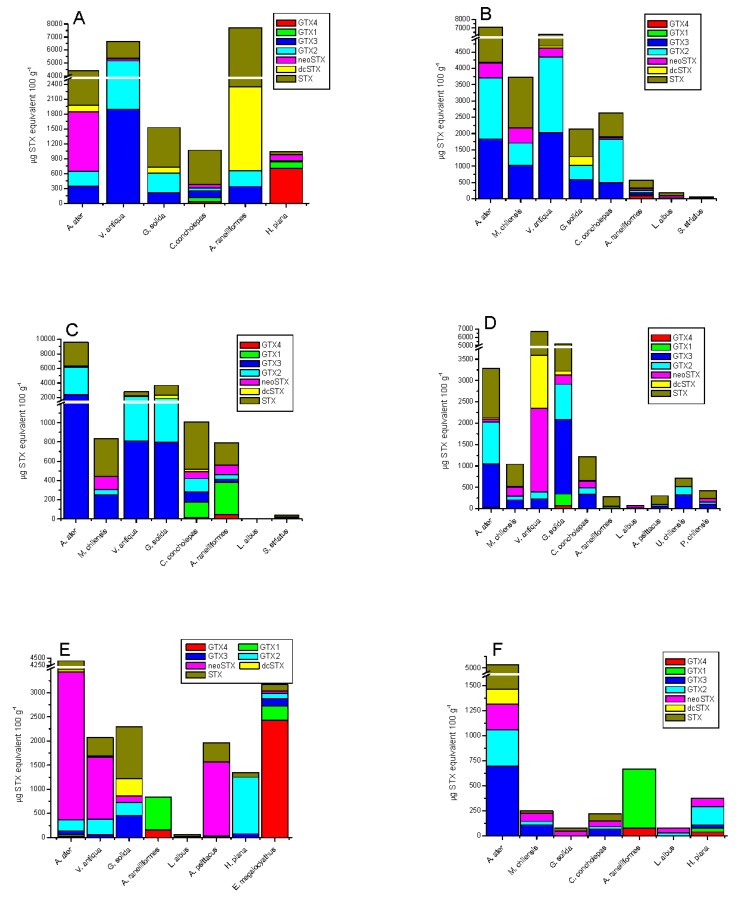
Comparison of STX-group toxin concentration of shellfish collected in the 6 extraction zones of marine vertebrates at Huichas Island, Aysén Region, Chile. Station 1 (**A**), station 2 (**B**), station 3 (**C**), station 4 (**D**), station 5 (**E**) and station 6 (**F**).

The frequency of toxic analogues identified in the different species was variable (Figure 4). In rocky strata-dwelling species, 15.4% for GTX3/GTX2, 4.8% for neoSTX, 3.3% for dcSTX and 58.8% for STX were detected; while in sandy bottom-dwelling species, 77.7% for GTX3/GTX2, 2.1% for neoSTX, 0.9% for dcSTX, and 19.3% for STX were detected (Figure 4A). In gastropods, variability was 11% for GTX4/GTX1, 17.5% for GTX3/GTX2, 7.1% for neoSTX, 0.4% for dcSTX, and 63.9% for STX (Figure 4B,C).; in crustaceans, variability was 80.5% for GTX4/GTX1, 2.2% for GTX3/GTX2, 11.6% for neoSTX, 0.1% for dcSTX, and 5.6% for STX (Figure 4C,D); in echinoderms: 6.3% for GTX3/GTX2, 44.9% for neoSTX, and 48.8% for STX (Figure 4E); in tunicates: 36% for GTX3/GTX2, 21.2% for neoSTX, 0.04% for dcSTX, and 23.9% for STX (Figure 4D); and finally, in octopus, it was 89% for GTX4/GTX1, 8.4% for GTX3/GTX2, 1.7% for neoSTX, and 4.2% for STX (Figure 4E). 

The toxicities detected in the different species collected at the different sampling points show that 95% of samples exceed the international limit (≤80 µg STX equiv 100 g^−1^). Nevertheless, species such as red sea urchin (echinoderms) showed toxicities close to the maximum allowed limit in some areas (>2.3%). In fish (*Salmo salar*), values were low (<LOD) and its toxicity was only found in the viscera, not in muscle. In cephalopods (southern red octopus, *Enteroctopus megalocyathus*), toxicity was exclusively dependent on the assessed tissue (distribution), where only toxins in fluids and visceras and neither in muscle nor in the nervous system were detected; these data correlate with those established in the literature [50]. Additionally, in the assessment of macroalgae *Macrocystis pyrifera*, the presence of toxins associated with the STX-group was not detected, so that the association of positive results through MBA to extracts of these microalgae could be associated to the presence of heavy metals such as Cadmium (Cd), Copper (Cu), Iron (Fe), Cobalt (Co), Lead (Pb), Silver (Ag), Aluminum (Al), and Arsenic (As); elements that, through adsorption and absorption in macroalgae, tend to produce false-positives results in the MBA [51,52].

It is noteworthy that in none of the evaluated areas, was the mortality of marine species (bivalves, gastropods and tunicates) detected, which correlates with the fact that species constantly exposed to HABs associated with STX-group toxins do not suffer negative effects on reduced filtration activity and absorption [53]. 

### 2.3. Tissue Distribution

**Figure 5:**
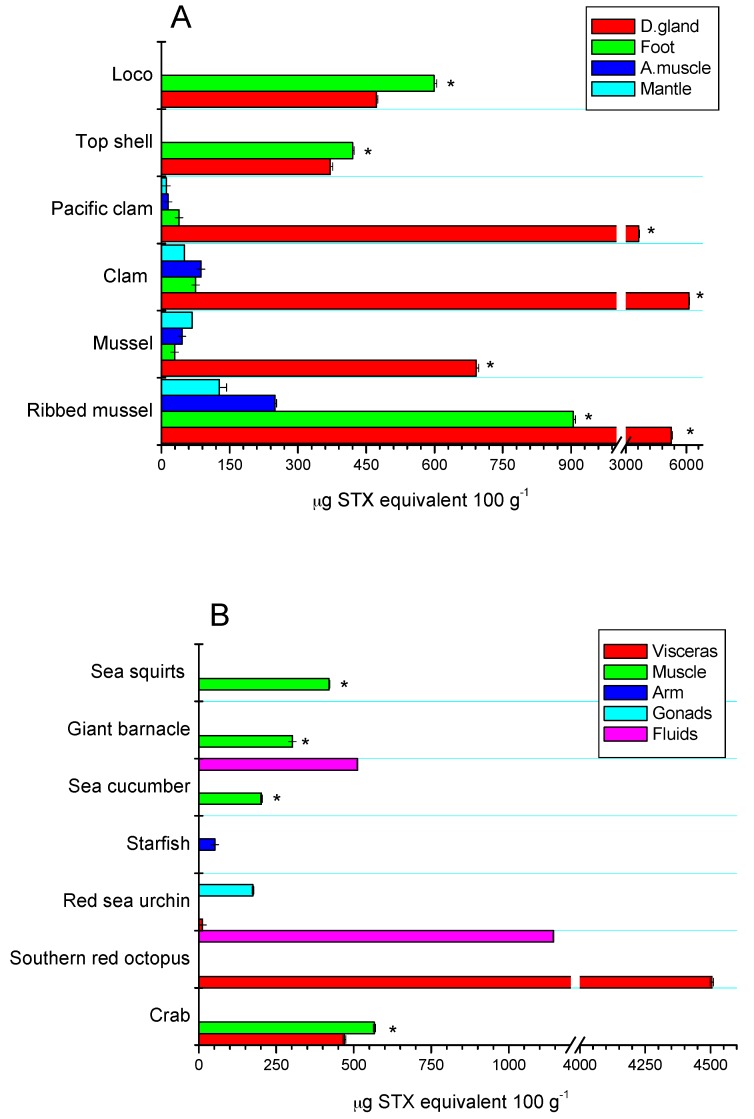
STX-group toxin concentration of marine organisms collected at the Huichas Island, Aysén Region. (**A**) Toxin concentration in edible parts (visceral and non-visceral tissues) of bivalves and gastropods; (**B**) toxin concentration in tissue of different unregulated marine vectors, collected in the study area.

The toxic distribution of the STX-group analogues in the different tissues of the assessed species was species-specific. In bivalves from rocky strata, the greatest toxic concentration was detected in the digestive glands (81.5%), adductor muscle (4.6%), mantle (5.3%), and foot (8.6%), highlighting the toxic concentration for ribbed mussel with 81% in digestive glands and 13.8% in the foot, with respect to the other tissues (*p* < 0.05) (Figure 5A). In sandy bottom-dwelling species, the toxic distribution was 97.4% in digestive glands, 1.0% in adductor muscle, 0.5% in mantle, and 1.1% in the foot. Furthermore, in gastropods (loco and top shell), toxic concentration was predominant in the foot (45.5%) and in the viscera (54.5%). These results are consistent with those obtained previously [7,49]. For the rest of the species, toxic concentration was predominantly detected in the muscle of the species (giant barnacle and sea squirts ≈98%), while for crabs and red sea urchin, the highest levels were detected in gonads with 55% and 94%, respectively; in southern red octopus and echiurans, the high levels of toxicity in viscera (80%) and fluids (71.7%) were highlighted, respectively (*p* < 0.05) (Figure 5B). 

The toxic profiles of dinoflagellates are variable and correlate directly with the state of the bloom development, emphasizing that in the cyst state, profiles are associated with thermodynamically more stable toxins (GTX1 and GTX2). Likewise, the high levels of STX-group analogues detected in digestive glands of seafood are directly related to the fact of being the first tissue where the compartmentalization of toxins occurs. Potentially, some physical and chemical elements tend to favor the first (enzymatic and non-enzymatic) transformations, producing the interconversion of analogues into thermodynamically more stable forms (β-isomers → α-isomers), in relation to what occurs in other non-visceral tissues [28,54]. Thus, the distribution of toxic analogues in other anatomical parts of the species (non-visceral tissue) is dependent on the species, which can be explained on the basis of the nutrient exchange in the system (clearance rate), favoring a greater distribution of the analogues in seafood with the consequent purification of toxins in the different species [7,55]. This toxic variability can be enhanced through the transfer of toxins through the trophic chain, allowing for the accumulation of toxins involved in other marine organisms (zooplankton and marine mammals) [50,56]. 

In fish (*S. salar*), the detection of toxins was scarce in the digestive system (<LOD) and neither toxins or damage in the tissue nor in the gills were detected. Gill damage detected in areas of blooms associated with *A. catenella* has been more linked to reactive oxygen species (ROS), docosahexaenoic acid (DHA) and potentially to other polyunsaturated fatty acids (PUFAs) [57]. However, mortality of fish (*S. salar*) was described in the area in the year 2009, a period in which cell density was ≈5000 cell mL^−1^. Notwithstanding, many reports indicate that the mortality of fish is not correlated with the number of cells associated with the bloom (LD_50_ oral in salmon ≈400–1000 µg STX equiv kg^−1^) [58,59].

### 2.4. Estimation of the Daily Intake 

Acceptable consumption intake according to the levels detected in the species showing low toxicity concentrations (red sea urchin and crabs) and close to the maximum allowed limit is shown in Table 1. In the red sea urchin, echiurans, and crabs, average values of 81.9, 714.2, and 2524.2 µg STX equiv 100 g^−1^ were detected, which represents an ingested dose of 5.4, 47.6, and 168.2 µg STX equiv kg^−1^ body weight respectively, when 400 g shellfish meat is consumed by a person of ≈60 kg body weight. Values > 3.5 times the lowest observed adverse effect level (LOAEL, 1.5 µg STX equiv kg^−1^ body weight), which can cause toxic effects on people [6], were detected in non-regulated products in Chile and which represent an important part of the diet through typical dishes such as crab pudding, sea urchin in green sauce, and stuffed echiurans. 

These toxic levels can be a risk for people and for higher-risk groups (<14 and >64 years-old), due to physiological variables that tend to emphasize the risk in these groups (pre-existing, high-risk diseases and gastrointestinal disorders). It must be emphasized that most of the assessed species show profiles with the higher toxic analogues (neoSTX, GTX3/GTX2 and STX) [60].

### 2.5. Food Implications

The Aysén region is the area where the largest number of natural banks of shellfish with high commercial value is found in Chile. However, since 1996, the area has tended to have been exposed to different types of HAB (*Dinophysis* sp., *Alexandrium* sp. and *Protoceratium* sp.), producing precautionary closures in the region due to the high toxic levels acquired by the different regulated shellfish species in the area. Previous studies have established that species can accumulate different types of toxins simultaneously (STX-group, OA-group, PTX-group, AZA-group, and YTX-group). However, the ranges of assimilation and distribution of each group of toxins is species-specific, which makes each species constitute a different food risk to the population in a different way, therefore, none of the species inhabiting an area with constant HAB should be underestimated.

The maximum concentration of STX-group toxins has been regulated worldwide (≤80 µg STX equiv 100 g^−1^), a regulation that is fulfilled in Chile [18]. However, some species are not considered in this regulation (red sea urchin, echiurans, crabs, and macroalgae) and ≈100% of those species are exported to European and Asian markets. In this study, some species did not exceed the standard, but they represent a high risk when the most toxic analogues corresponding to the STX-group are detected in their edible tissues (neoSTX, TEF = 2.0, and STX, TEF = 1.0).

The toxic variability in the different species from the resources collection areas can be explained based on the latitudinal salinity variables existing in the study area. Salinity ranges in the fjords strongly respond to the freshwater streams of the cordillera (mountain range), changes that may force physiological responses, such as a decrease in predation by gastropods or that some bivalves (*Mytilus chilensis)* may tend to lower their clearance and ingestion rates [39], which is reflected in prolonged periods of shellfish contamination. 

Intoxications produced by STX-group toxins have shown that toxins have a high metabolization rate towards glucuronide forms and towards more soluble analogues which, at the same time, are more toxic to human beings, which translates into a greater capacity for distribution of toxins in the tissues of the human body [20,61]. In this study, the total prevalence was 90% in bivalves, 50% in gastrops and giant barnacles, 15% in red sea urchin, 10% in crabs and sea cucumbers, and 5% in southern red octopus. The prevalence of toxins detected in shellfish was higher for GTX4/GTX1 in crustaceans (80.5%) and octopus (89%); GTX3/GTX2 in bivalves from sandy strata (77.7%); neoSTX in echinoderms (44.9%) and STX in gastropods (63.9%) and bivalves from rocky strata (58.8%)(*p* < 0.05). In addition, in all species, thermodynamically more stable analogues predominate in the ratio α > β epimers. These values coincide with those of Zamorano et al. (2013), establishing that the prevalence of STX-group toxins is higher in the spring-summer period [49]. 

In relation to the herbivorous species, *Fissurella nigra*, (whose diet is *Ulva* sp.) the toxicities detected are related to the toxin assimilation pathway through the feeding of periphyton (biofilm) which is often constituted of diatoms, cellular debris, and pseudofeces primarily, which contribute to a part of the toxicity detected [46].

In non-regulated species in Chile, such as crabs, N-sulfocarbamoyl-11-hydroxysulfate toxins were not detected, but stand out in all the profiles associated with the most toxic analogues (GTX3/GTX2, neoSTX and STX). This species (crabs)—characterized by being carnivorous and scavenging shellfish—accumulates toxins related to the STX-group from its prey, so, its total toxicity increases linearly with the amount of toxic mussels and crabs ingested by feeding, which transforms them into important vectors [62]. Even though the best bioindicator will always be mussels, crabs can maintain toxicity for a longer period of time [63]. Therefore, it is necessary to determine the toxicity per individual, considering that some Asian markets consume the entire specimen, hence, they are sold as a total individual.

In fish, the levels detected are low (LOQ), showing no apparent damage to the species or a real problem for the aquaculture. Even though the blooms are constant in the area, these have not been harmful to the larval stages of endemic fish in the region, either [64].

In Chile, currently the detection method for STX-group toxins is the MBA, which only determines the toxicity of the species, producing some false-positive results due to the interaction of the method with the presence of trace metals in algae and shellfish [51,65]. Even though HABs are constant in Chile (40–55° S) [34], there are few studies establishing the dynamics of toxic profiles associated in dinoflagellates and on the phases of contamination (assimilation, distribution and purification) of shellfish containing STX-group toxins. It should be noted that each species shows values characteristic for each phase with a prevalence of different toxic analogues in their tissues, which is translated into toxicities and variable toxic concentrations representing a risk to human health constantly. The per capita shellfish consumption in Chile is ~8.4 kg per year, which is a low value if compared to that from European and Asian countries, with 42 and 69 kg, respectively [66]. Nevertheless, this value tends to increase drastically during religious celebrations (Easter), a period that coincides with the highest prevalence of STX-group in ≈90% of the southern zone of Chile. This results in a high demand for marine resources, an increase in preventive analyses, and precautionary closures of contaminated areas. Even though there were a number of intoxicated people (23 people in 2016, with no casualties), the minimum levels to which people can be exposed to is ≈37 µg STX equiv 100 g^−1^ of tissue, a value corresponding to the cut-off of the MBA to detect STX-group toxins [6].

All of these assessed species stand out since they have a high dietary and commercial contribution, which makes them an important factor in the diet of some parts of the world and for a part of the population in Chile. The high variability detected in this study can be extrapolated to the population making up the natural banks and cultures in some species (mussels); due to this variability, in some species, toxicity can be overestimated when subjecting these contaminated resources to different cooking methods; processes that can favor the conversion of the analogues into more toxic forms (GTX4/GTX1 → neoSTX; GTX3/GTX2 → STX) [6,67].

In addition, the high toxic variability detected in all species shows either a direct (bivalves) or indirect (gastropods or limpets) assimilation of toxins associated with the STX-group, which is translated into toxic assimilation rates and variable detoxification rates over time. In the case of unregulated species (crabs, urchins, and octopus), these rates may be higher than those already established in species of bivalves and gastropods (≈10 months). This highlights the ignorance of the control authorities and the consumers, at a national and international level, of the toxic potential that certain species can acquire.

The high variability of toxicities detected with the STX-group in the different species is mainly due to: (1) the filtration capacity each species possesses; (2) variability of dinoflagellates in the water column (cell mL^−1^); (3) abiotic factors that interact directly with the physiology of the species and which are dependent on latitude, and (4) toxic transfer in the trophic chain which increases the variability and toxic predominance of the analogues related to the STX-group.

**Figure 6:**
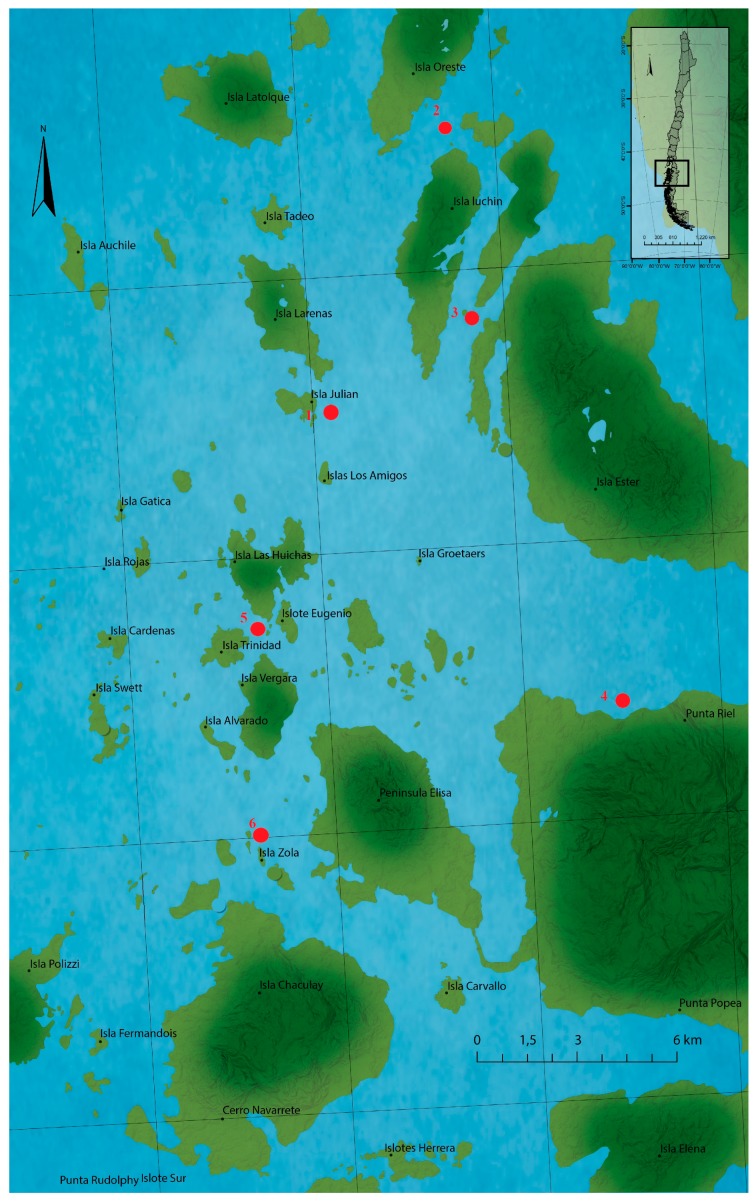
Locations of the sampling station at the Huichas Island, Chile. Red points represent the extraction zones of all marine resources.

Obtaining samples in the different austral zones (Figure 6), reveals that bivalves and gastropods are the main sources of the STX-group. All zones showed high toxicity levels with varying cellular levels in each zone, but always associated with *A. catenella*. The analysis of multiple evaluation points in microzones of all biological species exposed to HABs, associated with *A. catenella*, favor the acquisition of toxic concentrations more linked to the different species present in the extensive southern zone of Chile.

## 3. Conclusions

This is the first toxicological analysis covering all aquatic species, legislated or non-legislated, which have been exposed to HAB associated with the STX-group in Chile. Ninety-five percent of shellfish collected in areas of the region of southern Chile show high and variable levels of toxins associated with the STX-group. The most prevalent contaminated species were bivalves and gastropods, with the detection of highly toxic analogues (GTX3/GTX2, neoSTX, and STX). Toxicities of unregulated species in a sanitary manner (crabs and echiurans, *Fissurella* sp. and octopus) show a high risk for groups of extreme ages (<14 and >65 years). The identification in multiple vectors and in unregulated species is evidence that a risk assessment and risk management update are required and that the need to establish chemical and specific analyses for the detection of all analogues associated with the STX-group should be considered.

## 4. Materials and Methods

### 4.1. Chemical and Reagents

Methanol, acetonitrile, acetic acid, nitric acid, heptanesulfonic acid, hydrochlorid acid, peryodic acid, and peroxide hidrogene were purchased from Merck (Merck, Darmstadt, Germany). Deionizated water (<18 MΩ cm resistivity) was obtained from a MicroPure water purification system (Thermo scientific, Asheville, NC, USA). Chromatographic solvents were filtered through a membrane filter 0.45 µm from Merck (Merck Millipore Ltd., Cork, Ireland).

### 4.2. Standards

Analytic standards for toxin evaluation were acquired from the National Research Council Canada (Halifax, NS, Canada). To determine STX-group toxins, STX (CRM-STX-f), dcSTX (CRM-dcSTX), neoSTX (CRM-NEO-c), dcneoSTX (CRM-dcNEO), gonyautoxins (CRM-GTX2 & 3-c; CRM-GTX1 & 4-c; CRM-GTX5-b; CRM-dcGTX2 & 3-b), and C1–C2 (CRM-C1 & 2-b) were used. Stock solutions were diluted with acetic acid in order to obtain the appropriate work solutions. All solutions were stored in darkness at −20 °C.

### 4.3. Sampling 

Seven sampling points were selected along Huichas Island—Aysén Region (Chile)—in 2015 as shown in Figure 6 [7]; under authorization N° 1078 of the Regional Secretariat of Health Ministry, Aysén of Region, of the General Carlos Ibañez del Campo (Figure 6). All samples were manually collected and kept at −20 °C until analysis. 

The sites were chosen as part of a monitoring program for mussel densities and do not necessarily correspond to specific (or localized) sources of bloom. Sampling depths varied between 1 and 10 m, but the majority of samples were taken within the 2–8 m depth stratum. Samples were collected manually and stored at −20 °C until analysis. Plankton samples collected by vertical dragging showed the presence of *Alexandrium catenella*, identified by producing STX-group analogues [28,68].

Samples contained bivalves such as blue mussel (*Mytilus chilensis*), ribbed mussel (*Aulacomya ater*), clam (*Venus antiqua*) and Pacific clam (*Gari solida*), and gastropods such as loco (*Concholepas concholepas*) and top shell (*Argobuccinum ranelliformes*); sea star (*Stichaster striatus*), crabs (*Homalaspis plana*), red sea-urchin (*Loxechinus albus*), ascidian (Piure, *Pyura chilensis*), giant barnacle (*Austromegabalanus psittacus*), echiurans (*Urechis chilensis*), fissurellid limpet (*Fissurella nigra*), large brown algae (*Macrocystis pyrifera*), southern red octopus (*Enteroctopus megalocyathus*), and Atlantic salmon (*Salmo salar*).

For the analyses, five samples representative of each species were considered. Each sample included about 100 individuals per species, except for the *Concholepas concholepas* (*n* = 50), *Macrocystis pyrifera* (*n* = 15), *Enteroctopus megalocyathus* (*n* = 8) and *Salmo salar* which contained 10 selected individuals, prior to grouping the species in representative sizes for evaluation processing [7,49].

### 4.4. STX-Group Sample Preparation

Live native Chilean species were collected at Huichas Island upon arrival at the laboratory. Samples were extracted from shellfish separately, and 100 g of visceral (digestive glands) and non-visceral tissue (mantle, foot, and adductor muscle) was removed. The homogenized shellfish sample was then transferred to 250 mL centrifuge tubes with the same volume of 0.1 N HCl, and the toxins present in the samples were extracted following the AOAC procedure [69]. All samples were carefully treated to avoid variations in the profile of toxins produced by changes in pH. Small aliquots were taken to quantify the toxin concentration of the extracts by HPLC. Materials used during the experimental work were disposed of according to the normative for chemical and biological waste disposal of the Biosafety Unit of the Faculty of Medicine of the Universidad de Chile.

### 4.5. High Resolution Liquid Chromatography with Fluorescent Detection (LC-PCOX)

Detection of STX-group toxins was accomplished by using the LC-PCOX AOAC 2011.02 technique [70]. An HPLC unit (Young Lin Instrument, Co., Anyang, Korea) was used, equipped with a binary pump (YL9101) at a constant flux of 0.8 mL/min of the mobile phase, with a Rheodyne 7725i (loop 20 μL) coupled to a spectrofluorometric detector (FP-2020 Plus, Jasco, Tokio, Japan), in an excitation range of 330 nm and an emission range of 390 nm. To determine carbamate toxins (GTXs and STX), a 3.5 μm reverse phase C-8 column (Zorbax Bonus-RP, 4.6 × 150 mm, Agilent Technologies Co., Ltd., Santa Clara, CA, USA) was used and, to determine sulfocarbamoyl toxins (C1/C2) a 5 μm reverse phase C-8 column (BetaBasic-8, 4.6 × 250 mm, Fisher Scientific, Nepean, ON, Canada) at constant 37 °C (column compartment YL 9131, YL Instrument Co., Ltd. Gyeonggi-do, Korea). Besides the LC binary pump, two additional isocratic pumps (YL9200) (YL Instrument Co., Ltd. Gyeonggi-do, Korea) were used, one with an oxidant agent and the other with 500 mM acetic acid, with a flux of 0.4 mL/min. All elements were on line inside a reaction oven at 85 °C (CO-IV Scienhome, Scienhome Scientific Instrument Co. Ltd., Tianjin, China), which contained a 10 m, coiled peek tubing with a total volume of 1 mL for derivatization of toxins. All toxins were identified comparing their retention time (R_t_) measured as min/V. Quantification of each analog was done according its 0.01 to 4.5 µg interval of STX-equivalent (*r^2^* = 0.9989) calibration curve. LOD and LOQ of STX-equivalents was calculated according IUPAC criteria, establishing a range between 0.005 up to 0.02 µg g^−1^ and 0.01 up to 0.2 µg g^−1^ respectively [7,15,70]. Total toxicities of species were expressed as µg STX-equivalent 100 g^−1^, utilizing the TEF of each toxin [6].

### 4.6. Method Validation

The inter-day precision and accuracy of the method were determined by analyzing three different concentrations over five days. Intra-day accuracy and precision were calculated from six repeat injections. The LOD (S/N = 3:1) and LOQ (S/N = 10:1) were calculated from standard chromatograms [71]. 

### 4.7. Statistical Analyses

Results were expressed as mean ± SEM (*n* = 5). Calibration curves were obtained through regression analyses. Differences between groups were analyzed using one or two-way analysis of variance (ANOVA) depending on the number of variables to analyze. A *p* < 0.05 significance level was considered for all cases. Analyses were performed using GraphPad Prism software (GraphPad Prism 7, GraphPad Software, Inc., La Jolla, CA, USA).

## Figures and Tables

**Table 1:** The saxitoxin-group (STX-group) toxin concentration in edible parts of marine organisms collected from Huichas Island, Aysén of Region and dose of STX equivalents ingested by a person from 400 g of shellfish.

Species	μg STX eq 100 g^−1^	Dose of STX Equiv Ingested by a Person of 60 kg from 400 g of Shellfish (μg STX equiv/kg Body Weight)	ARfD * μg STX equiv/kg Body Weight
*Venus antiqua*	>1900 ± 7.3	126.6	0.5
*Gari solida*	>180 ± 1.2	12.0	0.5
*Aulacomya ater*	>4100 ± 5.1	273.3	0.5
*Mytilus chilensis*	>1500 ± 2.6	100.0	0.5
*Argobuccinum ranelliformes*	>300 ± 1.1	20.0	0.5
*Concholepas concholepas*	>129.0 ± 2.1	8.6	0.5
*Homalaspis plana*	2524 ± 4.1	168.2	0.5
*Loxechinus albus*	81.9 ± 0.6	5.4	0.5
*Pyura chilensis*	420.0 ± 2.4	28	0.5
*Austromegabalanus psittacus*	>301.1 ± 0.7	20.1	0.5
*Urechis chilensis*	714.2 ± 3.5	47.6	0.5
*Fissurella nigra*	15.0 ± 0.1	1.0	0.5
*Enteroctopus megalocyathus*	n.d.	---	0.5
*Salmo salar*	n.d.	---	0.5
*Macrocystis pyrifera*	n.d.	---	0.5

* Acute reference dose, n.d. = none detected.
